# Combined Effects of Halloysite Nanotubes, Nucleating Agent, and Thermal Annealing on the Printability and Mechanical Performances of 3D-Printable Polypropylene Random Copolymer-Based Composites

**DOI:** 10.1021/acsomega.6c03068

**Published:** 2026-06-10

**Authors:** Boonlom Thavornyutikarn, Kawinthip Inthana, Wasana Kosorn, Paanrapee Hongsaprapart, Wanida Janvikul, Kalyanee Sirisinha

**Affiliations:** † Biofunctional Materials and Devices Research Group, 61191 National Metal and Materials Technology Center, National Science and Technology Development Agency, Pathum Thani 12120, Thailand; ‡ School of Materials Science and Innovation, Faculty of Science, 26685 Mahidol University, Nakhon Pathom 73170, Thailand; § Department of Chemistry, Faculty of Science, Mahidol University, Bangkok 10400, Thailand; ∥ Materials and Design Unit for Medical Devices and Healthcare, Faculty of Science, Mahidol University, Bangkok 10400, Thailand

## Abstract

This study developed three-dimensional (3D)-printable polypropylene random copolymer (PPR)-based composites incorporating halloysite nanotubes (HNTs) and a nucleating agent (NA) for extrusion-based additive manufacturing. The combined effects of HNT loading (0–5 wt %), NA incorporation (0.1 wt %), and postprinting thermal annealing on their thermal behavior, crystallization, printability, and mechanical performance were systematically investigated. Thermogravimetric analysis (TGA) demonstrated enhanced thermal stability with HNT addition due to the barrier effect of the aluminosilicate structure. Differential scanning calorimetry (DSC) revealed that HNTs had limited influence on crystallization behavior, whereas NA significantly increased the crystallization and onset temperatures, indicating accelerated crystallization kinetics without altering the dominant α-phase crystal structure, as confirmed by X-ray diffraction (XRD). HNT incorporation improved filament dimensional stability during extrusion, yielding uniform, near-circular cross sections compared to neat PPR, and enhanced build plate adhesion and dimensional stability during printing without external adhesives. Mechanically, HNT addition increased stiffness but reduced impact strength. Postprinting thermal annealing increased melting enthalpy and refined crystalline morphology, resulting in partial recovery of impact strength in HNT-filled samples, although the values remained below that of neat PPR. The combined use of HNT incorporation, nucleation control, and thermal annealing provided an effective strategy to enhance the 3D-printability and mechanical performance of PPR-based composites.

## Graphical abstract

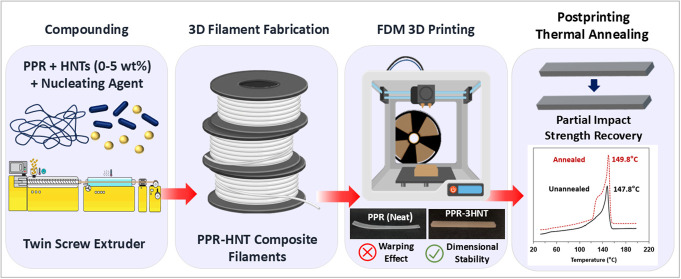

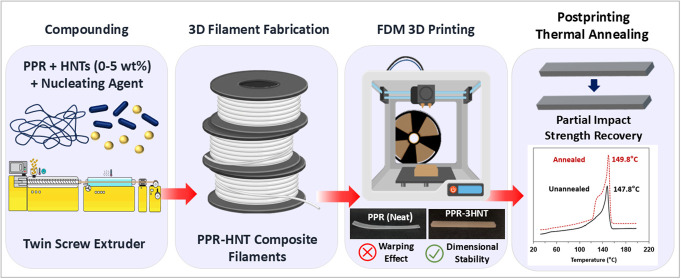

## 1 Introduction

Fused deposition modeling (FDM) has emerged as a widely adopted extrusion-based additive manufacturing technique capable of fabricating complex geometries directly from digital models. Owing to its cost-effectiveness, design flexibility, and operational simplicity, FDM has evolved from a rapid prototyping tool to a manufacturing platform for functional components in industries including healthcare, aerospace, and automotive engineering.^1–3^ Despite these advantages, the mechanical performance and dimensional accuracy of FDM-printed parts remain highly dependent on material behavior during extrusion and cooling. Currently, most commercial FDM filaments are based on polymers such as polylactic acid (PLA), acrylonitrile butadiene styrene (ABS), and thermoplastic polyurethane (TPU) which exhibit low shrinkage and good dimensional stability.^4,5^ Although PLA is inherently semicrystalline, its slow crystallization kinetics under typical FDM processing conditions often result in predominantly amorphous printed structures, thereby minimizing shrinkage-related deformation. In contrast, semicrystalline thermoplastics with faster crystallization and higher volumetric contraction, while attractive for their superior chemical resistance, fatigue performance, and thermal stability, remain underutilized in FDM due to crystallization-induced warpage and dimensional instability.

Polypropylene (PP) is a widely used semicrystalline polyolefin valued for its low density, chemical inertness, mechanical robustness, recyclability, and cost-effectiveness.^6^ However, its application in FDM is limited by crystallization-driven shrinkage, which leads to severe warpage and dimensional inaccuracy. The high crystallinity and volumetric contraction of PP during cooling generate internal stresses that promote deformation and build plate detachment.^7^ In addition, the intrinsically low surface energy of PP reduces interlayer bonding and platform adhesion during printing.^4,8^

Polypropylene random copolymer (PPR), containing small amounts of ethylene or other α-olefin comonomers, exhibits reduced crystallinity and lower volumetric contraction compared to PP homopolymer, making it more suitable for extrusion-based additive manufacturing.^7^ Nevertheless, shrinkage-induced deformation and insufficient dimensional stability remain significant challenges. Therefore, effective regulation of crystallization behavior and melt stability is still required to improve the printability and structural integrity of FDM-printed PPR components.

Incorporation of inorganic fillers into polymer matrices has emerged as a promising strategy to enhance stiffness, thermal stability, and dimensional control in FDM filaments.^9–12^ Nanofillers such as carbon nanotubes,^9^ talc,^10^ and layered silicates^11^ have been shown to alter crystallization behavior and restrict polymer chain mobility, thereby improving structural rigidity and reducing deformation during cooling. Among these fillers, halloysite nanotubes (HNTs) have attracted increasing attention due to their naturally occurring tubular morphology, high aspect ratio, and aluminosilicate composition. HNTs possess a double-layered structure and provide mechanical reinforcement while acting as physical barriers to thermal degradation.^12,13^ Their geometry and interfacial interactions enable stress transfer within polymer matrices, making them promising candidates for lightweight and thermally stable PP-based composites. However, systematic investigations of HNT-filled polypropylene systems for FDM, particularly using polypropylene random copolymer, remain limited, and the influence of HNT incorporation on filament dimensional stability and printability has not been comprehensively explored.

Beyond reinforcement, controlling crystallization is crucial for minimizing shrinkage-induced stress in semicrystalline polymers. Nucleating agents (NAs) promote heterogeneous nucleation, accelerate crystallization kinetics, and refine crystalline morphology, thereby influencing spherulite size, lamellar organization, and residual stress development.^14–16^ While nucleating agents are extensively applied in conventional processing methods such as injection molding, their integration into FDM-printable polypropylene systems, particularly in combination with nanofillers, has not been systematically investigated.

Postprinting thermal annealing represents an additional strategy to tailor crystalline structure and relieves internal stresses. Annealing below the melting temperature enables molecular rearrangement and structural reorganization, which can enhance dimensional stability and mechanical performance in FDM-printed parts.^17–19^ However, the combined effects of filler reinforcement, nucleation control, and postprocessing annealing on the structural evolution and performance of polypropylene random copolymer-based composites have not yet been comprehensively studied.

Therefore, this work systematically investigated the combined effects of halloysite nanotubes (HNTs), a nucleating agent (NA), and postprinting thermal annealing on the crystallization behavior, filament quality, dimensional stability, and mechanical performance of 3D-printable PPR-based composites. By correlating nanofiller incorporation, crystallization regulation, and thermal treatment with printability-related performance, this study established an integrated strategy for enhancing the structural reliability and functional performance of PPR in extrusion-based additive manufacturing.

## 2 Materials and Experiments

### 2.1 Materials

Polypropylene random copolymer (coded as PPR) with a melt flow index of 8 g/10 min was purchased from HMC Polymers Co., Ltd., Thailand, and employed as the primary matrix phase in this study. Halloysite nanotubes (coded as HNTs) were supplied by Sigma-Aldrich Corporation and used as an inorganic nanofiller in the composite system. HNTs exhibited a tubular morphology, with an average diameter and length of approximately 80 and 500 nm, respectively, as demonstrated by transmission electron microscopy (TEM) imaging (Figure S1, Supporting Information). Hyperform HPN-68L, a dicarboxylate sodium-based compound supplied by Milliken Co. USA, was used as a nucleating agent (coded as NA).

### 2.2 Preparation of PPR-HNT Composites

The preparation of PPR-HNT compounds and the fabrication of composite specimens are illustrated in Scheme 1. Prior to compounding, the HNT particles were dried at 80 °C overnight to remove the moisture entrapped within the material. A masterbatch (MSB) containing 10 wt % HNTs in the PPR matrix was prepared by melt-compounding in a twin-screw extruder (LTE16-40, Labtech Engineering Co., Ltd., Samut Prakan, Thailand) with a temperature profile ranging from 120 to 190 °C. The extrudate was cooled, pelletized, and used as a concentrated intermediate.

**Scheme 1:**
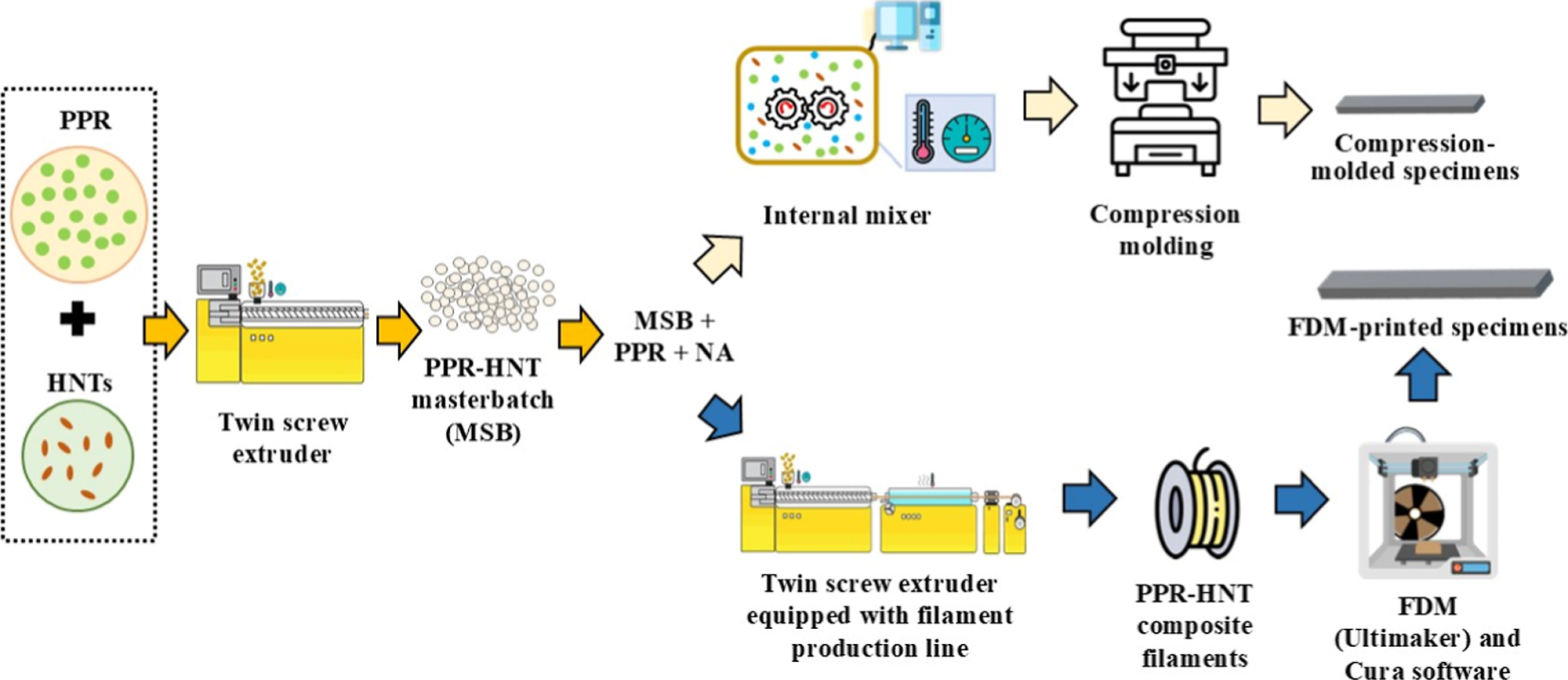
Schematic Illustration of the Preparation Process of the PPR-HNT Composite Specimens

The masterbatch was subsequently diluted with virgin PPR to obtain composite formulations containing 0, 1, 3, 5, and 7 wt % HNTs, designated as PPR100, PPR-1HNT, PPR-3HNT, PPR-5HNT, and PPR-7HNT, respectively. A fixed amount of a nucleating agent (NA, 0.1 wt % based on the total mass of the PPR-HNT composite) was incorporated into all the composites. PPR-HNT composites without NA addition were also prepared for comparison and denoted by the superscript “#” (i.e., PPR100^#^, PPR-1HNT^#^, PPR-3HNT^#^, PPR-5HNT^#^ and PPR-7HNT^#^).

All the formulations were compounded in an internal mixer (Haake Rheocord 90, Haake, Germany) equipped with cam-type rotors at 170 °C, 20 rpm, for 17 min. The compounded materials were then cooled to room temperature, granulated, and compression-molded at 200 °C under 10 MPa for 5 min, followed by cooling to ambient temperature under pressure using a hydraulic press (Carver Inc., USA).

The actual HNT content in each composite formulation was determined by the ash content measurement according to ASTM D7348 ^20^ with some modifications. Briefly, 2 g of each composite sample was placed in a porcelain crucible and subjected to thermal oxidation in a temperature-programmed electric furnace. The samples were heated at 500 °C for 1 h, followed by heating at 750 °C for 1 h, and then held at 750 °C for an additional 2 h to ensure complete combustion and volatilization of the polymer matrix. The furnace was then cooled to room temperature, and the crucibles were weighed to determine the residual mass. The remaining residue in the crucibles, derived from the incorporated HNT filler in the sample, was used to calculate the filler loading. Each measurement was performed in duplicate (*n* = 2), with the results summarized in Table 1, along with the nominal compositions and corresponding sample codes.

**Table 1:** Compositions of the PPR-HNT Composites Prepared in This Study

sample code	PPR (wt %)	HNT (wt %)	NA (wt %)	actual HNT content (%)
MSB	90	10	-	8.41 ± 0.07
PPR100	100	0	0.1	-
PPR-1HNT	99	1	0.1	1.51 ± 0.11
PPR-3HNT	97	3	0.1	2.99 ± 0.06
PPR-5HNT	95	5	0.1	4.41 ± 0.02
PPR-7HNT	93	7	0.1	5.78 ± 0.03

### 2.3 Preparation of PPR-HNT Filaments

The PPR-HNT filaments were fabricated by melt-blending using a modular 16 mm twin-screw extruder equipped with a filament production line and a 3 mm die (LTE16-40, Labtech Engineering Co., Ltd., Samut Prakan, Thailand), as illustrated in Scheme 1. The barrel temperature profile ranged from 115 to 185 °C, while the die temperature of the gear pump was maintained between 170 and 180 °C, and the screw rotation speed was fixed at 180 rpm. The molten composites were extruded through a die into a water bath maintained at 80 °C using a pulling speed of 5 m/min to form continuous filaments which were subsequently wound onto a spool using a filament rolling system.

The extrusion parameters for each composition were optimized to achieve stable filament dimensions. The diameter of each fabricated filament was measured at 50 cm intervals along the filament length using a digital caliper. The average filament ovality was determined by measuring the maximum and minimum diameters of the cross-sectional area of each extruded filament at 20 randomly selected locations of the filament, according to eq 1.^21^

1 $$Ovality=\frac{1}{20}\sum_{n=1}^{n=20}\left(D_{\max}-D_{\min}\right)$$

where *D*_max_ and *D*_min_ denote the maximum and minimum diameters (mm), respectively, and *n* is the measurement location along the filament.

### 2.4 FDM 3D Printing of PPR-HNT Composite Filaments

FDM 3D printing of each PPR-HNT composite filament was performed using an FDM 3D printer (Ultimaker 3, Geldermalsen, The Netherlands) equipped with a glass build plate. Ultimaker Cura slicing software (version 5.7, Ultimaker, Geldermalsen, The Netherlands) was used to convert the STL models into G-code files based on computer-aided design (CAD) models for printing. The optimized 3D printing parameters for all the prepared composite filaments are summarized in Table 2, using a raster angle of 45°/-45° in all printing processes.

**Table 2:** Operating Parameters for FDM 3D Printing of the PPR-HNT Composite Filaments

operating variable	setting value
nozzle temperature (°C)	220
printing bed temperature (°C)	100
nozzle diameter (mm)	0.8
layer thickness (mm)	0.25
printing speed (mm/s)	30
print infill (%)	100
raster angle	45°/-45°
chamber temperature (°C)	25°

### 2.5 Thermal Annealing Treatment

Both compression-molded specimens and 3D-printed articles were subjected to thermal annealing in a convection oven at 120 °C for 30, 60, and 360 min. After annealing, the specimens were cooled naturally to room temperature under ambient conditions prior to further characterizations.

### 2.6 Characterizations

#### 2.6.1 Thermogravimetric Analysis (TGA)

TGA was carried out using a TGA 2 STARe System (Mettler Toledo, USA) to evaluate the thermal stability of the PPR-based composites. The samples were heated from 30 to 600 °C under a nitrogen environment, followed by heating from 600 to 800 °C under an oxygen environment at a rate of 10 °C/min. The TGA thermograms were recorded, and the characteristic degradation temperatures were evaluated, including the temperature at 5% weight loss (T_5_), the temperature at 10% weight loss (T_10_), and the maximum degradation temperature (*T*_max_).

#### 2.6.2 Differential Scanning Calorimetry (DSC)

The DSC experiment was conducted using a Q200 model DSC instrument (TA Instruments, Newcastle, DE, USA) to evaluate the thermal properties of neat PPR and its corresponding composites containing various concentrations of HNTs. Samples weighing 8–10 mg were first heated to 200 °C under a nitrogen atmosphere, then cooled to 25 °C at a rate of 20 °C/min, and finally heated to 200 °C at the same rate. The melting temperature (*T*_m_), crystallization temperature (*T*_c_), onset temperature of crystallization (*T*_c onset_), and the melting enthalpy (Δ*H*_m_) were determined.

#### 2.6.3 X-Ray Diffraction (XRD) Analysis

X-ray diffraction patterns of the neat PPR and the PPR-HNT composites were obtained using an X-ray powder diffractometer (JEOL, JDX-3530 Theta-2Theta), equipped with a copper radiation source (CuKα, λ = 0.1542 nm). The PPR-HNT composite specimens, with dimensions of 8 mm × 8 mm × 2 mm (width x length x thickness), were fabricated from the composite compounds. XRD measurements were performed at an operating voltage of 40 kV and a current of 30 mA, over an angular range (2θ) of 5°–40°, with a scanning rate of 0.04°/sec.

#### 2.6.4 Scanning Electron Microscopy (SEM)

The morphological characteristics of the PPR-HNT composites were examined using a field emission scanning electron microscope (FE-SEM, HITACHI/SU5000, Japan). The samples were cryogenically fractured in liquid nitrogen to expose their internal structures, and the fractured surfaces were then sputter-coated with a thin layer of gold (approximately 15 nm in thickness) and observed at an accelerating voltage of 5 kV. The characteristic of the chemical composition of the PPR-HNT composite was carried out using energy dispersive spectroscopy (EDX) using INCA (Oxford Instruments) software.

#### 2.6.5 Shrinkage and Build Plate Adhesion Measurement

The dimensional shrinkage and adhesion between printed specimen and FDM build plate of each PPR-HNT composite were evaluated to assess its dimensional stability and interfacial bonding during printing.

Shrinkage was determined by comparing the external dimensions of each printed specimen with the corresponding CAD dimensions (10 mm × 80 mm × 4 mm), as described in eq 2. The width, length, and thickness of each specimen were measured using a digital caliper (Mitutoyo 500-196-30) with a resolution of 0.01 mm and an accuracy of 0.02 mm. The percentage shrinkage in each dimension and the volumetric shrinkage were calculated as follows

2 $$Volumetric\;shrinkage\left(\%\right)=\frac{V_{CAD}-V_{printed}}{V_{CAD}}\times 100$$

where *V*_CAD_ and *V*_printed_ represent the designed and measured volumes of the specimen, respectively.

Build plate adhesion was qualitatively evaluated by visually assessing the contact adhesion between the printed specimen and the glass build platform after fabrication. Rectangular specimens with dimensions of 10 mm × 80 mm × 4 mm (width x length x thickness) were fabricated from each PPR-HNT composite filament, using the processing parameters listed in Table 2. Neither adhesive nor additional surface treatment was applied to the glass build platform, ensuring that the observed adhesion behavior reflected the intrinsic material-substrate adhesion. Afterward, the FDM-printed specimens on the build plate were cooled to room temperature. The presence or absence of curling, upward bending, or corner detachment of the specimens was visually inspected, and representative photographs were taken to qualitatively illustrate the adhesion behavior.

#### 2.6.6 Water Contact Angle Measurement

The surface wettability of each 3D-printed specimen obtained from Section 2.6.5 was assessed through water contact angle measurement using the sessile droplet method on a goniometer (Ramé-hart model 250 Goniometer, USA) at ambient temperature. A 2 μL droplet of distilled water was deposited onto the specimen surface via an automatic microsyringe. The droplet profiles were captured after 5 s using a high-speed video camera and subsequently analyzed with Dropimage software (Ramé-hart Instrument Co., USA). Five measurements were conducted on different positions for each specimen (*n* = *5*), and the determined water contact angles of the individual as-printed specimens were presented as the mean ± SD.

#### 2.6.7 Mechanical Property Evaluation

The mechanical performances of the 3D-printed specimens were evaluated through flexural and Izod impact tests. Flexural properties of the FDM-printed specimens (10 mm × 80 mm × 4 mm; width x length x thickness) were measured in accordance with ISO 178 ^22^ using a Universal Testing Machine (AGX-V, Shimadzu, Japan) equipped with a 10 kN load cell. The tests were conducted under a three-point bending configuration at room temperature, with a support span of 64 mm and a crosshead speed of 2 mm/min.

Notched Izod impact strength of the printed specimens with identical dimensions was determined according to ISO 180 ^23^ using a Pendulum Impact Tester (GT-7045-MDH) fitted with a 2.75 J hammer using an impact velocity of 3.46 m/s. At least five independent specimens fabricated from the same PPR-HNT composite filament were analyzed for each test.

### 2.7 Statistical Analysis

A statistical analysis of the mechanical properties of the FDM-printed composite specimens was performed using SPSS software (version 31.0; SPSS, Inc., Chicago, IL). A one-way analysis of variance (ANOVA), followed by Duncan’s multiple range posthoc test, was used to determine statistically significant differences among the sample groups. Data were obtained from five independent specimens (*n* = 5) and presented as the mean ± standard deviation (SD). Differences were considered statistically significant at *p* values < 0.05.

## 3 Results and Discussion

### 3.1 Effects of HNTs and NA Addition on the Properties of PPR-Based Composites

#### 3.1.1 Thermal Stability and Degradation Behavior

The thermal stability and degradation behavior of the PPR-based composite compounds were investigated by thermogravimetric analysis (TGA) to ensure that the materials possessed sufficient thermal stability for the fabrication of 3D-printed objects. This analysis also verified that the incorporation of HNTs and NA did not adversely affect the thermal stability of the PPR matrix in the composites.

Figure 1 presents the derivative thermogravimetric analysis (DTG) curves of all the composite samples. The corresponding degradation temperatures (T_5,_ T_10_, and *T*_max_) are summarized in Table 3. Neat PPR (PPR100) exhibited a characteristic single-step thermal degradation behavior between 390 and 500 °C, typical of polypropylene thermal decomposition.^24^ Similarly, all the PPR-HNT composites displayed a single-step degradation profile, indicating that the incorporation of HNTs did not alter the fundamental degradation mechanism of the PPR matrix in the composites.

**Figure 1:**
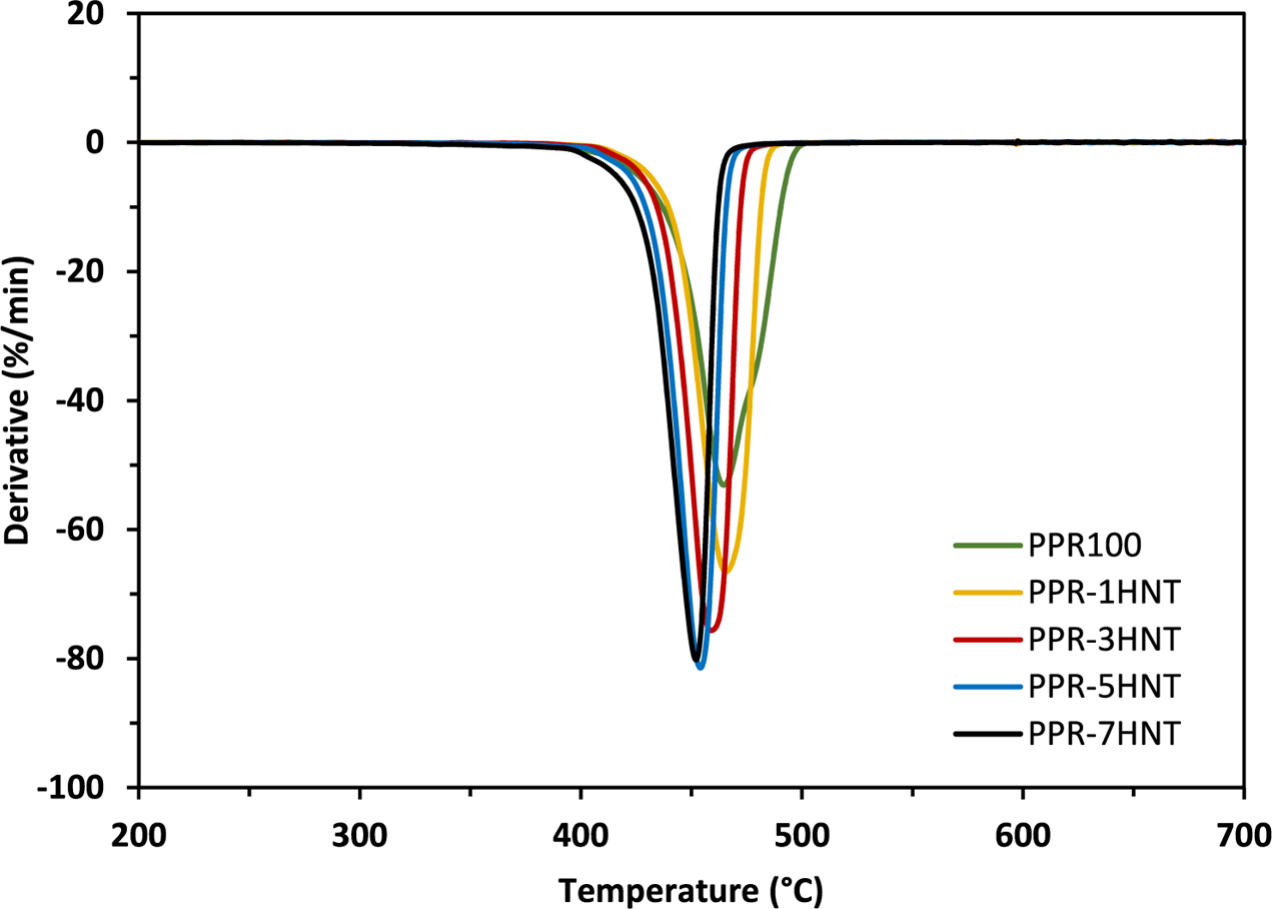
DTG thermograms of the PPR-based composites being heated from 50 to 800 °C.

**Table 3:** TGA Results of the Neat PPR and PPR-HNT Composite Compounds

sample code	T_5_ (°C)	T_10_ (°C)	*T*_max_ (°C)
PPR100	395.9	421.9	464.5
PPR-1HNT	434.8	444.3	465.5
PPR-3HNT	431.7	439.8	458.9
PPR-5HNT	425.1	434.2	454.0
PPR-7HNT	414.6	427.5	452.0

The addition of small amounts of HNTs noticeably delayed the onset of thermal degradation of the composites, as evidenced by the shift of the T_5_ and T_10_ values toward higher temperatures (Table 3). These upward shifts were particularly pronounced at low HNT loadings (PPR-1HNT and PPR-3HNT). The incorporation of 1 to 5 wt % HNTs increased the T_5_ temperature by approximately 30 °C or more. At low HNT contents, a good dispersion of less agglomerated HNT particles in the PPR matrix acted as a rigid physical barrier, restricting heat transfer and hindering the diffusion of volatile degradation products, thereby delaying polymer chain scission during thermal decomposition.^25^ Nonetheless, a slight decrease in T_5_ and T_10_ values was observed at a higher HNT loading of 7 wt %. This could be ascribed to the partial agglomeration of HNT particles, which diminished the effectiveness of the heat barrier and limited interfacial contact with the PPR matrix, consequently lowering the thermal stabilization performance.

Considering the *T*_max_ values, PPR-1HNT composite exhibited a *T*_max_ value comparable to the neat PPR; however, higher HNT loadings resulted in a progressive shift toward lower temperatures. This observed behavior in the main degradation phase, notwithstanding the initial stability, was explicitly associated with two main effects: the impairment of the heat-barrier efficiency due to HNT agglomeration, and the catalytic degradation of the PPR matrix facilitated by the increased concentration of surface hydroxyl (Al–OH) groups at elevated temperatures.^26^ This observation was in accordance with a previous study demonstrating that incorporating high HNT loadings into the linear low-density polyethylene (LLDPE) matrix could create localized heat sink sites, facilitating accelerated polymer degradation.^27^ However, all the composite formulations exhibited higher T_5_ and T_10_ than neat PPR, reflecting their suitability for filament preparation and FDM-3D printing processes.

To further investigate the dispersion and distribution of HNTs in the composite formulations, scanning electron microscopy (SEM) analysis was conducted on the cryogenically fractured surfaces. As illustrated in Figure 2, the cryo-fractured surface of neat PPR exhibited a relatively smooth and featureless morphology, characteristic of brittle fracture behavior,^28^ with no discernible evidence of plastic deformation. After the incorporation of HNTs, the SEM micrographs of the PPR-HNT composite specimens revealed bright and glossy features embedded within the PPR matrix (Figure 2b–d), corresponding to the presence of HNT particles (indicated by yellow arrows). These features originated from the higher electron-scattering contrast of aluminosilicate nanotubes, thereby confirming successful incorporation of the nanofiller in the PPR matrix.^25^ Furthermore, the presence of HNTs was corroborated by EDX analysis (as shown in Figure S2), which clearly revealed the main elements of O, Si and Al. The absence of voids associated with filler pull-out suggested that the HNT particles were satisfactorily embedded within the PPR matrix.

**Figure 2:**
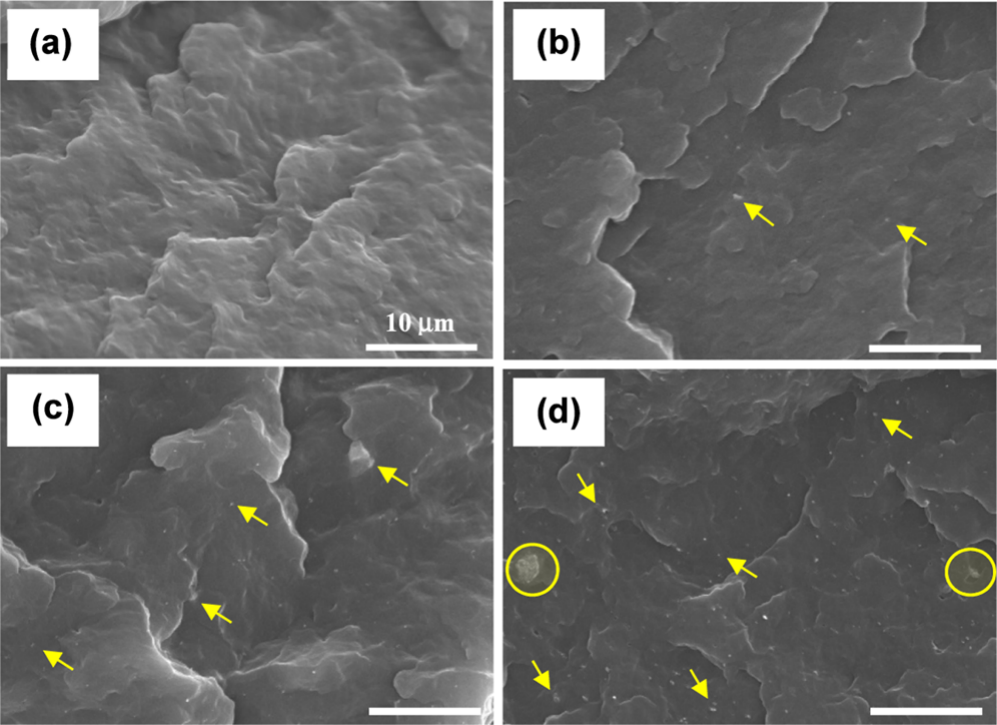
SEM micrographs of the cryo-fractured surfaces of (a) PPR100, (b) PPR-1HNT, (c) PPR-3HNT, and (d) PPR-5HNT composites at a magnification of 3000×. Yellow arrows indicate the presence of HNT particles, while yellow circles highlight localized HNT agglomerates in the PPR-HNT composites.

At lower HNT loadings (1 to 3 wt %), a uniform particle distribution of HNTs was observed, indicating effective compounding of HNTs and PPR, while at a higher loading of 5 wt % HNTs, the SEM micrograph (Figure 2d) revealed the presence of localized HNT agglomerates (indicated by yellow circles), suggesting a gradual reduction in dispersion homogeneity at the elevated filler content. This phenomenon arose primarily from the high surface energy of HNTs and the presence of silanol groups (-Si–OH) on the outer HNT surfaces, promoting interparticle hydrogen bonding.^29^

While the SEM results qualitatively showed changes in dispersion with increasing HNT contents, the thermal degradation behavior was not solely governed by agglomeration. Instead, the TGA results, particularly the shifts in T_5_, T_10_ and *T*_max_, were more appropriately explained by a combination of barrier and catalytic effects, as stated above.

#### 3.1.2 Crystallization Behavior

Figure 3 presents the differential scanning calorimetry (DSC) results of the PPR-HNT composites with and without the addition of a nucleating agent (NA). The PPR-based composites containing HNT loadings of 1 to 5 wt % were examined because a higher HNT loading (7 wt %) exhibited reduced thermal stabilization efficiency and was less effective as a filler. To ensure that the crystallization behavior of PPR in all formulations was solely influenced by the presence of HNTs and NA, the *T*_m_ and Δ*H*_m_ values reported in Figure 3 were obtained from the second heating of the DSC thermograms.

**Figure 3:**
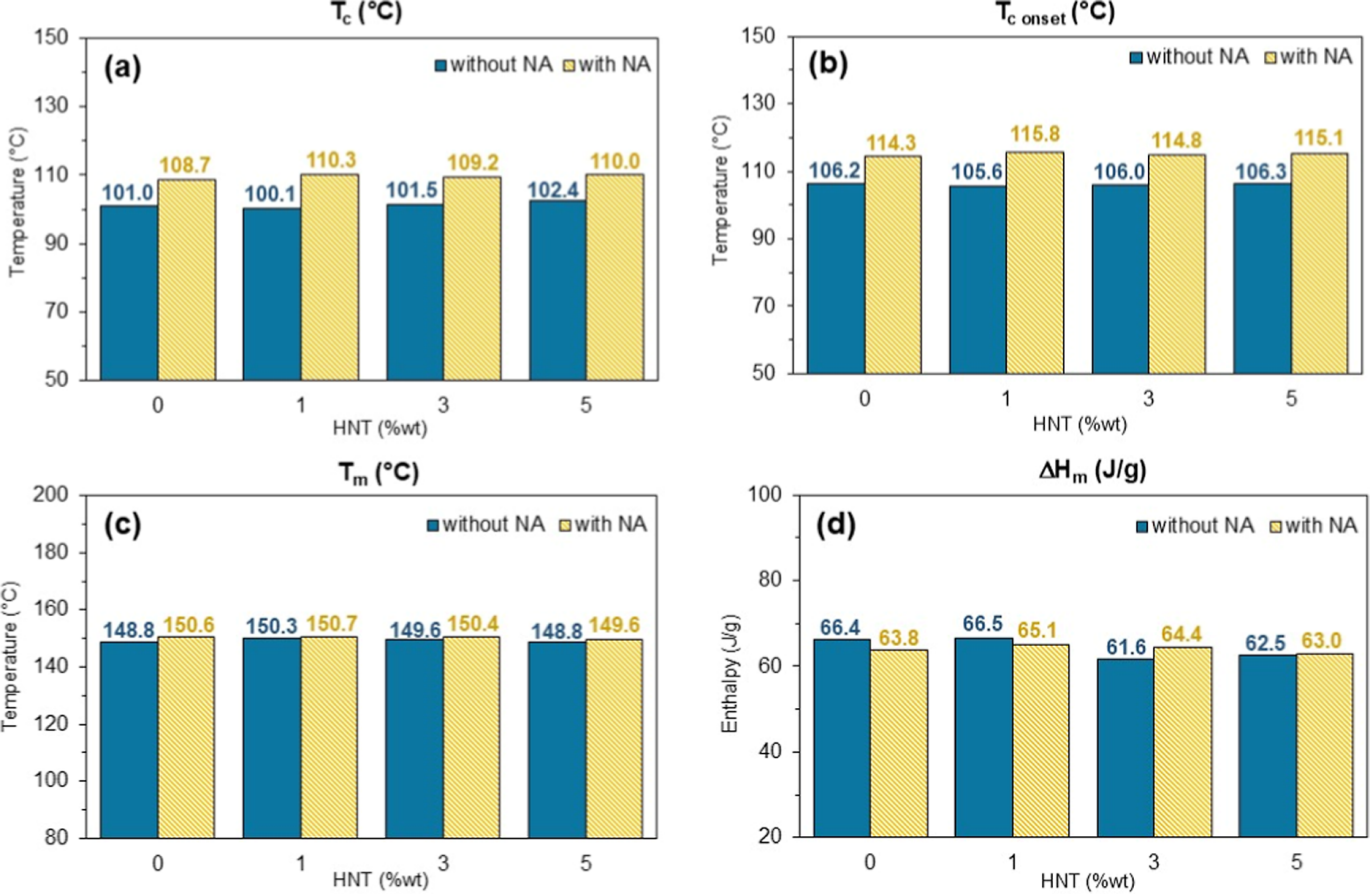
Thermal properties of the PPR-HNT composites with and without a nucleating agent. (a) *T*_c_, (b) *T*_c onset_, (c) *T*_m_, and (d) Δ*H*_m_ of PPR-HNT composites containing 0 to 5 wt % HNTs.

Neat PPR exhibited a crystallization temperature (*T*_c_) of approximately 101.0 °C, an onset of crystallization temperature (*T*_c onset_) of 106.2 °C, a melting temperature (*T*_m_) of 148.8 °C, and an enthalpy of melting (Δ*H*_m_) of 66.4 J/g. These values were consistent with the typical thermal behavior of PPR reported in the literature.^30^

Overall, the incorporation of HNTs exhibited a minor effect on the crystallization behavior of the PPR composite matrix. With the incorporation of HNTs at 1 to 5 wt %, only slight variations in *T*_c_ and *T*_c onset_ were observed. *T*_c_ remained within a narrow range of 100.1 °C–102.4 °C, while *T*_c onset_ varied marginally from 105.6 to 106.3 °C. Δ*H*_m_, which was related to the overall degree of crystallinity, changed slightly with increasing HNT content. Furthermore, the *T*_m_ of all the composite formulations remained nearly constant at 150 °C, indicating that the crystalline structure and lamellar stability of PPR were largely preserved within this nanofiller concentration range.

In contrast, the addition of NA to PPR had a pronounced effect on the crystallization behavior of PPR, as evidenced by a marked shift of both *T*_c_ and *T*_c onset_ of PPR to higher temperatures. The *T*_c onset_ of PPR increased from 106.2 to 114.3 °C, confirming the high nucleating efficiency of Hyperform HPN-68L by promoting PPR crystallization, while the *T*_c_ of PPR increased by approximately 8 °C after the addition of NA. Compared to the *T*_c_ and *T*_c onset_, *T*_m_ and Δ*H*_m_ changed slightly. After the incorporation of a nucleating agent into the PPR-HNT composite systems, the higher *T*_c_ and *T*_c onset_ values were consistently observed across all HNT loadings, with increases of 7 to 10 °C relative to their NA-free counterparts. The increase in *T*_c_ and *T*_c onset_ upon the addition of nucleating agents is commonly observed in polypropylene systems,^31–34^ indicating enhanced heterogeneous nucleation and accelerated crystallization kinetics. In contrast, *T*_m_ and Δ*H*_m_ exhibited only marginal changes, suggesting that the nucleating agent primarily increased nucleation density without significantly altering lamellar thickness or the ultimate crystallizable fraction of the copolymer. Similar behavior was reported in the literature.^31–34^ For example, the addition of a comparable α-nucleating agent, Hyperform HPN-68L (0.25 wt %), resulted in only a minor increase in the crystallinity of isotactic polypropylene (∼3%), while *T*_m_ remained essentially unchanged.^31^

Overall, all the composite formulations demonstrated melting temperatures similar to that of neat PPR, suggesting that the same processing conditions could be applied for both PPR and the composites. Moreover, the higher *T*_c onset_ promoted by the addition of NA in the composites was advantageous for FDM-based 3D printing, enabling crystallization to take place at a higher temperature upon cooling, which could beneficially improve the dimensional stability of the printed parts.^35^

### 3.2 Effect of Thermal Annealing Treatment on the Crystallization of PPR-Based Composites

Thermal annealing is widely employed as an effective postprocessing approach to modify the crystallization kinetics and crystalline structure in semicrystalline polymers. Table 4 shows the *T*_c_, *T*_c onset_, *T*_m_, and ΔH_m_ values of the compression-molded composite specimens before and after annealing at 120 °C for various durations. Data from the unannealed samples (0 min) were also collected for comparison. Clearly, thermal annealing significantly influenced the crystallization behavior of neat PPR and the PPR-HNT composites. Compared to the unannealed samples, the annealed counterparts exhibited increased *T*_c onset_, *T*_c_, and Δ*H*_m_ values, accompanied by a slight decrease of *T*_m_ values, indicating that the materials were characterized by a greater number of smaller spherulites. This observation was further supported by the DSC thermograms shown in Figure 4, displaying the first heating curves of the PPR-based samples before and after a 360 min annealing. It was evident that all the annealed samples displayed changes in melting behavior, compared to that of their unannealed counterparts. In particular, the appearance of a shoulder on the melting peak indicated modifications in the crystalline structure. This shoulder, occurring at a lower temperature than the main melting peak, reflecting the presence of smaller or less perfect crystalline domains that melted at lower temperatures.

**Table 4:** Crystallization Temperature (*T*_c_), Onset Temperature of Crystallization (*T*_c Onset_), Melting Temperature (*T*_m_), and Melting Enthalpy (Δ*H*_m_) of PPR-Based Composites with and Without the Incorporation of a Nucleating Agent, Before and After Thermal Annealing

sample code	*T*_c_ (°C)				*T*_c onset_ (°C)				*T*_m_ (°C)				Δ*H*_m_ (J/g)			
	0 min	30 min	60 min	360 min	0 min	30 min	60 min	360 min	0 min	30 min	60 min	360 min	0 min	30 min	60 min	360 min
PPR-HNT Composites Without A Nucleating Agent																
PPR100^#^	101.0	105.0	105.1	105.1	106.2	109.6	109.4	109.2	150.0	145.7	145.9	147.2	67.3	71.3	71.7	74.7
PPR-1HNT^#^	100.1	107.0	106.1	106.4	105.6	110.3	109.1	109.9	151.9	146.2	146.0	148.0	69.0	70.7	69.1	72.3
PPR-3HNT^#^	101.5	103.7	104.4	106.2	106.0	109.5	110.6	109.6	152.3	146.6	146.3	147.8	59.6	65.3	69.4	71.3
PPR-5HNT^#^	102.4	105.5	105.4	104.8	106.3	110.0	110.4	110.3	150.7	146.3	146.3	147.6	62.4	66.2	67.6	69.0
PPR-HNT Composites with A Nucleating Agent																
PPR100	108.7	115.2	115.4	115.3	114.3	119.6	119.7	119.4	152.2	149.0	148.1	148.5	62.6	67.5	69.6	71.1
PPR-1HNT	110.3	112.9	113.2	112.4	115.8	117.2	117.6	117.0	152.0	146.9	147.8	149.4	62.6	68.9	71.8	70.1
PPR-3HNT	109.2	112.0	112.1	111.7	114.8	116.3	116.5	116.6	150.0	147.3	147.5	149.1	62.6	67.2	69.6	70.5
PPR-5HNT	110.0	112.0	111.8	111.4	115.1	116.4	116.4	116.0	149.9	146.8	147.5	148.3	63.7	65.1	69.2	69.1

**Figure 4:**
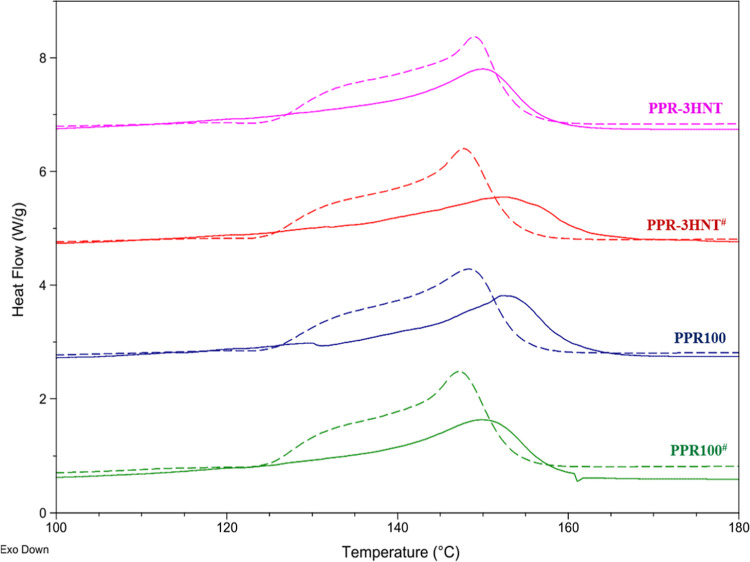
DSC first heating curves of PPR-based samples before (solid lines) and after (dashed lines) annealing at 120 °C for 360 min.

As shown in Figure 4 and Table 4, the effect of annealing on the crystallization of PPR was more pronounced in the presence of a nucleating agent. After 30 min annealing, the *T*_c onset_ of PPR increased from 114.3 to 119.6 °C. The additional nucleation sites were introduced by the nucleating agent, promoting the formation of a finer spherulitic morphology with a higher nucleation density. As a result, these smaller spherulites melted at lower temperatures, leading to a slight decrease in *T*_m_ (149.0 °C). Varying the annealing time between 0 and 360 min had minimal impact on the *T*_c_, *T*_c onset_, and *T*_m_ values. Conversely, the Δ*H*_m_ increased with increasing annealing time. For example, neat PPR (PPR100) annealed in the presence of NA for 360 min exhibited the Δ*H*_m_ value of 71.1 J/g compared to 62.6 J/g of the unannealed PPR sample. Increasing the annealing time allowed the polymer chains to reorganize and form a denser crystal structure of higher crystallinity.

In the composite system containing 1 to 5 wt % HNTs, the influence of a nucleating agent was less pronounced than that in the unfilled PPR. The presence of HNTs hindered an increase in *T*_c_ and *T*_c onset_ of the PPR-HNT composites observed after annealing, with the effect becoming more significant at higher HNT loadings. A similar trend was also detected in melting behaviors, where a reduction of *T*_m_ and Δ*H*_m_ in the annealed composites was recorded when the HNT loading was increased. The presence of HNTs introduced a constrained interfacial region that restricted polymer chain mobility, thereby limiting chain diffusion and reducing the ability of chains to reorganize into ordered crystalline structures.^27,36,37^ In the systems containing a nucleating agent, although nucleation was promoted, the restricted chain mobility imposed by HNT counteracts subsequent crystal growth. This effect became more pronounced at higher HNT loadings and consequently suppressed the increase in *T*_c_ and *T*_c onset_, as crystallization was governed not only by nucleation but also by chain rearrangement and crystal growth. As a result, the reduction in Δ*H*_m_ was less significant in nucleated composites compared to that of non-nucleated systems, even after annealing. For instance, after 360 min of annealing, Δ*H*_m_ decreases from 74.7 (PPR100^#^) to 69.0 J/g (Δ = 5.7 J/g) in the unnucleated system containing 5 wt % HNT (PPR-5HNT^#^), whereas a smaller decrease from 71.1 (PPR100) to 69.1 J/g (PPR-5HNT) (Δ = 2.0 J/g) was observed in the corresponding nucleated composite.

X-ray diffraction (XRD) analysis was, apart from DSC, employed to investigate the crystalline phase structures of the PPR-based composite materials. Both neat PPR (PPR100) and NA-containing PPR-HNT compression-molded specimens were analyzed before and after thermal annealing at 120 °C for 30 min. The XRD results are presented in Figure 5. The neat PPR sample (PPR100) displayed diffraction peaks at 2θ values of 13.7°, 16.3°, 18.2°, and 21.4°, which were characteristic of the α-monoclinic crystalline form of polypropylene, and corresponded to the (110), (040), (130), and (111/-131) crystallographic planes, respectively.^38^ These characteristic reflections indicated that α-phase crystallization was predominant in both unannealed and annealed PPR.

**Figure 5:**
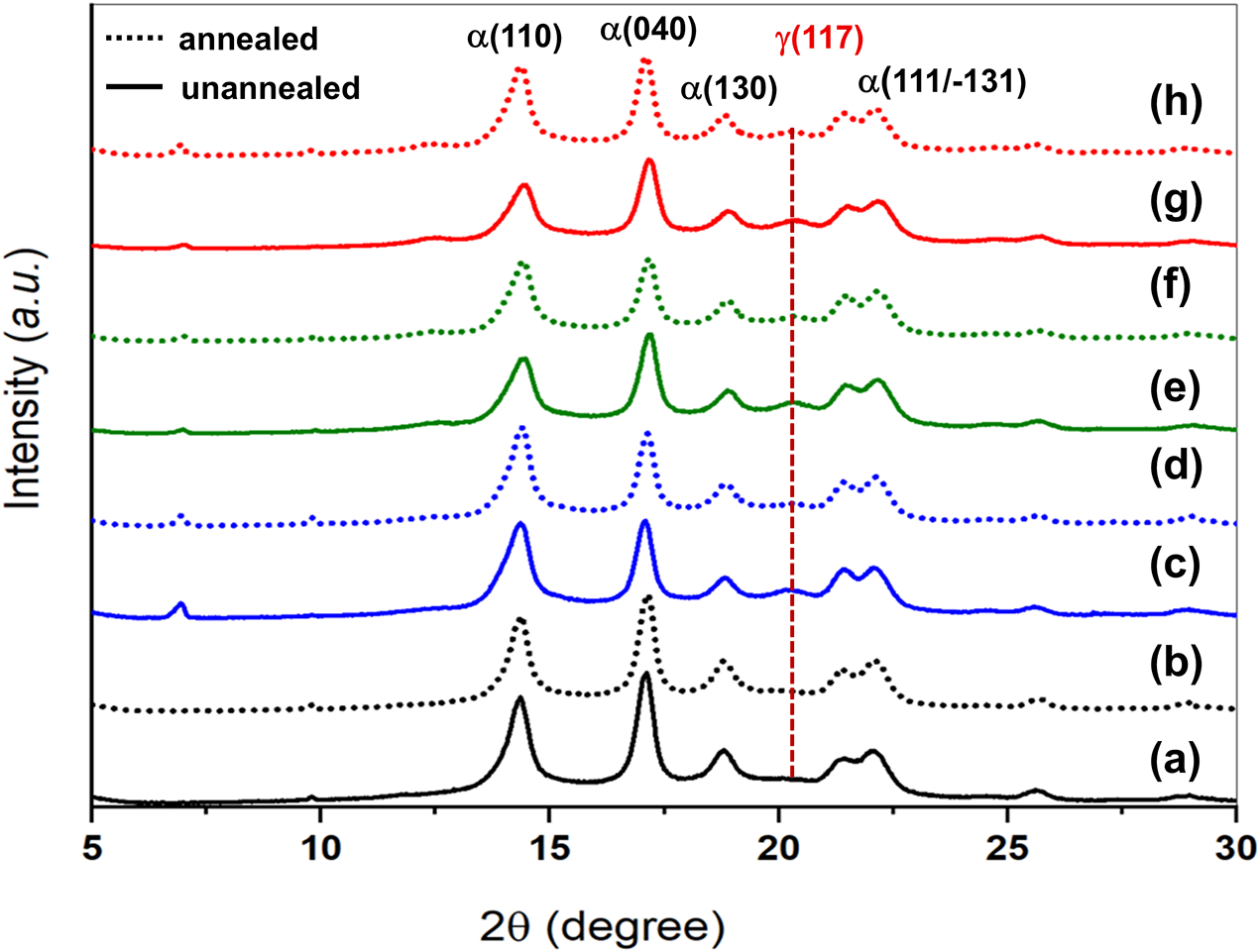
X-ray diffraction (XRD) patterns of (a,b) PPR100, (c,d) PPR-1HNT, (e,f) PPR-3HNT, and (g,h) PPR-5HNT before (solid lines) and after (dashed lines) thermal annealing at 120 °C for 30 min.

The PPR-HNT composites with HNT loadings (1 to 5 wt %) displayed diffraction patterns comparable to that of neat PPR, suggesting that the primary crystalline structure of the PPR composite matrix was still preserved. Moreover, no diffraction peak associated with the β-crystalline form, typically observed around 16°, was detected in any of the test samples,^39^ indicating that the incorporation of HNTs did not induce a β-crystalline phase transformation of the PPR matrix.

A weak diffraction feature was also observed in the composites at 2θ ∼ 20°. This reflection was attributed to the (117) crystallographic plane of the γ-phase polypropylene^40^ or the (100) reflection of halloysite nanotubes (PDF Card #29-1487), typically appearing at 2θ ∼ 20.1°.^41^ The γ-phase of polypropylene exhibited a relatively low melting temperature and transformed to the thermodynamically more stable α-form on heating, particularly in low molecular weight samples crystallized under conditions of high undercooling.^42^ However, the low intensity of this diffraction features relative to the dominant α-phase reflections suggested that the crystalline structure of the composites remained predominantly α-phase.

Importantly, a slight difference in the XRD patterns of the unannealed and annealed composites was observed; slightly increased intensities of the diffraction peaks were detected, indicating that the thermal treatment under the selected conditions promoted a modest enhancement in crystalline order and/or crystallinity, rather than inducing a change in crystalline phase. These XRD observations were consistent with the DSC results, demonstrating that the nucleating agent primarily modified the crystallization kinetics by increasing the onset temperature of crystallization, while maintaining the α-phase as the dominant crystalline structure of the PPR matrix.

### 3.3 Physical Properties of 3D Printable PPR-HNT Composite Filaments

Consistent diameter and roundness are crucial characteristics of successful filament fabrication, as this directly impacts FDM printability. To ensure reliable printability, the extruded filaments must possess a smooth surface, uniform diameter, and well-controlled roundness to maintain a constant and continuous flow through the heated nozzle.^43^ In this study, PPR-HNT composite filaments containing both HNTs and NA were extruded with a targeted average diameter of 2.85 mm. The dimensional accuracy and roundness of the fabricated filaments were evaluated by measuring the difference between the maximum and minimum diameters of the filament. The ovality was then calculated according to eq 1, as described in Section 2.3, to assess their suitability as feedstocks for FDM printing. The average filament diameters and corresponding ovality values are summarized in Table 5. All the fabricated PPR-HNT composite filaments exhibited smooth surfaces (as illustrated in Figure S3) with average diameters ranging from 2.75 ± 0.03 to 2.79 ± 0.03 mm, which were slightly lower than the targeted diameter. This deviation was attributed to the volumetric shrinkage of the PPR matrix during cooling and solidification. The low standard deviations indicated a stable extrusion process, yielding filaments with sufficient dimensional uniformity for subsequent FDM printing.

**Table 5:** Average Diameters and Ovality Values of the Extruded PPR-HNT Composite Filaments

filament code	average diameter (mm)	average ovality (mm)
PPR	2.75 ± 0.03	0.14 ± 0.04
PPR-1HNT	2.75 ± 0.03	0.10 ± 0.03
PPR-3HNT	2.78 ± 0.03	0.07 ± 0.04
PPR-5HNT	2.73 ± 0.03	0.07 ± 0.04
PPR-3HNT^#^	2.79 ± 0.02	0.07 ± 0.03

The average ovality values, reflecting filament roundness, ranged from 0.14 mm for neat PPR filament to 0.10–0.07 mm for PPR-HNT composite filaments. The neat PPR filament exhibited a higher ovality value, indicating a greater deviation from circularity and reduced dimensional uniformity, while the incorporation of HNTs significantly improved filament roundness by approximately 30–50% (reduced ovality values). The explicitly lower ovality values of the composite filaments indicated more consistent cross-sectional geometry, suggesting that HNT incorporation enhanced filament dimensional stability during extrusion. This was plausibly attributed to enhanced melt elasticity and restricted chain mobility, arising from interfacial confinement and physical interactions between the HNTs and the polymer chains.^10,44^ Notably, most of HNT-incorporated PPR filaments exhibited nearly identical ovality values, indicating that low HNT loadings (1–5 wt %) were sufficient to enhance PPR melt flow behavior and promote filament dimensional stability. Moreover, both NA-free and NA-added PPR-3HNT filaments had almost the same ovality values, suggesting that the increased filament dimensional stability was governed by the incorporation of HNT rather than NA.

### 3.4 Printability of 3D-Printable PPR-HNT Composite Filaments

Adequate adhesion between the first deposited layer and an FDM build plate is a prerequisite for successful 3D printing, as insufficient adhesion can lead to warpage or premature detachment during fabrication. The evaluation of bed adhesion of the PPR-based composite filaments was conducted without the use of any adhesive or fixation spray. The 3D specimens of PPR100, PPR-1HNT, PPR-3HNT, PPR-5HNT, and PPR-3HNT^#^ were individually fabricated by printing the corresponding filaments layer-by-layer onto a glass build platform using the printing parameters listed in Table 2. To assess interfacial adhesion and printability, the representative printing images were captured and illustrated in Figure S4. The results indicated that the 3D articles could not be successfully printed from all of the prepared composite filaments. During fabrication, the as-printed PPR100 and PPR-1HNT parts exhibited pronounced shrinkage, leading to noticeable edge lifting and detachment from the glass platform prior to print completion. Such a printing phenomenon was attributed to weak interfacial adhesion arising from the inherently low surface energy of polypropylene, which limits its adhesion to common build plate materials such as glass.^8^ In contrast, the PPR-3HNT, PPR-5HNT, and PPR-3HNT^#^ articles were successfully printed on the glass build plate, demonstrating relatively greater build plate adhesion. The enhanced bed adhesion of these composite filaments was positively attributed to the incorporation of a sufficient amount of polar HNT particles, which increased the overall surface polarity of the composites. This was confirmed by a gradual decrease in water contact angles of the 3D-printed samples integrated with increasing HNT content: 101.28° ± 1.09°, 100.20° ± 2.16°, 95.98° ± 1.08°, 93.98° ± 0.75°, and 95.44° ± 1.92° for PPR100, PPR-1HNT, PPR-3HNT, PPR-5HNT, and PPR-3HNT^#^, respectively (see the optical images of water droplets in Figure S5). Such a trend rather agreed with a previous report indicating that the incorporation of HNTs enhanced the wettability of ultrahigh molecular weight polyethylene (UHMWPE)-based nanocomposite films.^45^ In addition, HNT incorporation partially restricted the crystallization of PPR, thereby reducing shrinkage-induced internal stress during cooling, and further contributing to improved build plate adhesion upon printing.

Figure 6 displays the digital photographs of the 3D composite articles. The as-printed PPR-3HNT and PPR-3HNT^#^ specimens exhibited stable adhesion throughout the printing process without warpage, indicating an optimal balance between melt flow behavior and shrinkage control. As anticipated, the as-printed PPR-5HNT specimen also showed good adhesion and shape retention after printing. These observations apparently suggested that the incorporation of 3–5 wt % HNT could sufficiently improve the dimensional stability of the articles being fabricated.

**Figure 6:**
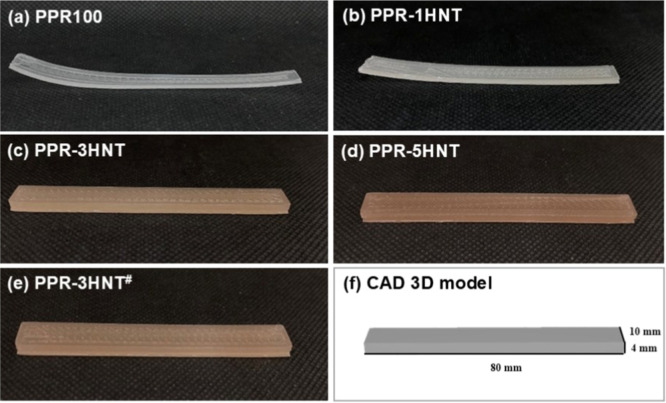
Digital photographs of 3D specimens fabricated from (a) PPR100, (b) PPR-1HNT, (c) PPR-3HNT, (d) PPR-5HNT, and (e) PPR-3HNT^#^, taken after being removed from the glass build surface. An image from a CAD model (f) displays the 3D specimen dimensions.

The volumetric shrinkages of the printed specimens are summarized in Table 6. The shrinkage values for the neat PPR and PPR-1HNT objects were not reported, as both compositions could not be successfully printed due to severe warping and early detachment from the build plate, which prevented the fabrication of intact specimens for reliable measurement. This behavior reflected their poor dimensional stability under the current processing conditions. By contrast, the PPR-3HNT, PPR-5HNT, and PPR-3HNT^#^ parts were successfully printed and exhibited consistent volumetric shrinkage of approximately 14% with low variability, indicating their good dimensional stability. For comparison, polypropylene random copolymer (PPR), composed of a small amount of ethylene or α-olefin comonomers, has been reported to exhibit up to 36% volumetric contraction under conventional processing conditions.^7^ The substantially lower shrinkage observed in the present study suggested that HNT incorporation effectively mitigated crystallization-induced contraction during cooling. A comparable shrinkage behavior was observed for both PPR-3HNT and PPR-3HNT^#^ printed specimens, suggesting that the addition of the nucleating agent did not provide a further improvement in the dimensional stability of the composites under the FDM printing conditions employed. HNT incorporation solely played a primary role in controlling material shrinkage during printing.

**Table 6:** Volumetric Shrinkage of FDM-Printed PPR-HNT Composite Articles

sample code	measured specimen height (experimental) (mm)	height retention (%)^a^	%shrinkage (vol)^b^
PPR100	1.07 ± 0.17	ND	ND
PPR-1HNT	1.77 ± 0.23	ND	ND
PPR-3HNT	3.42 ± 0.01	85.42 ± 0.14	14.58 ± 0.14
PPR-5HNT	3.42 ± 0.01	85.58 ± 0.29	14.42 ± 0.29
PPR-3HNT^#^	3.42 ± 0.02	85.50 ± 0.50	14.50 ± 0.50

a Calculated relative to the nominal CAD dimension (Figure 6f).

b Calculated from a volume change (eq 2) and expressed as the mean ± SD (*n* = *5*).

ND: Not determined due to incomplete 3D specimen fabrication.

### 3.5 Mechanical Performances of 3D-Printed PPR-HNT Composite Specimens

Individual 3D specimens (dimensions of 10 mm (width) × 80 mm (length) × 4 mm (thickness)) were fabricated from neat PPR and PPR-HNT composite filaments using the printing parameters summarized in Table 2. A small amount of adhesive was applied on the glass build plate to enhance the bed adhesion of the printed filaments. The adhesive residue was subsequently wiped off with Kimwipes soaked with ethanol prior to the mechanical test. Figure 7 shows the effects of HNT loading and postprinting thermal annealing (at 120 °C for 360 min) on the flexural and impact performances of the 3D-printed PPR-based composite specimens. Figure 7a and b reveal that the flexural modulus of the as-printed specimens increased with increasing HNT content, whereas the flexural strength remained largely unchanged. This trend was attributed to the inherent rigidity of the HNT particles and their ability to restrict the mobility of PPR molecular chains, resulting in enhanced stiffness without significantly improving stress transfer at the polymer–filler interface. Overall, postprinting thermal annealing did not produce notable and definite changes in both flexural strength and modulus, compared with those of the as-printed counterparts, suggesting that the applied annealing conditions were insufficient to substantially modify the crystalline load-bearing structure governing flexural deformation.

**Figure 7:**
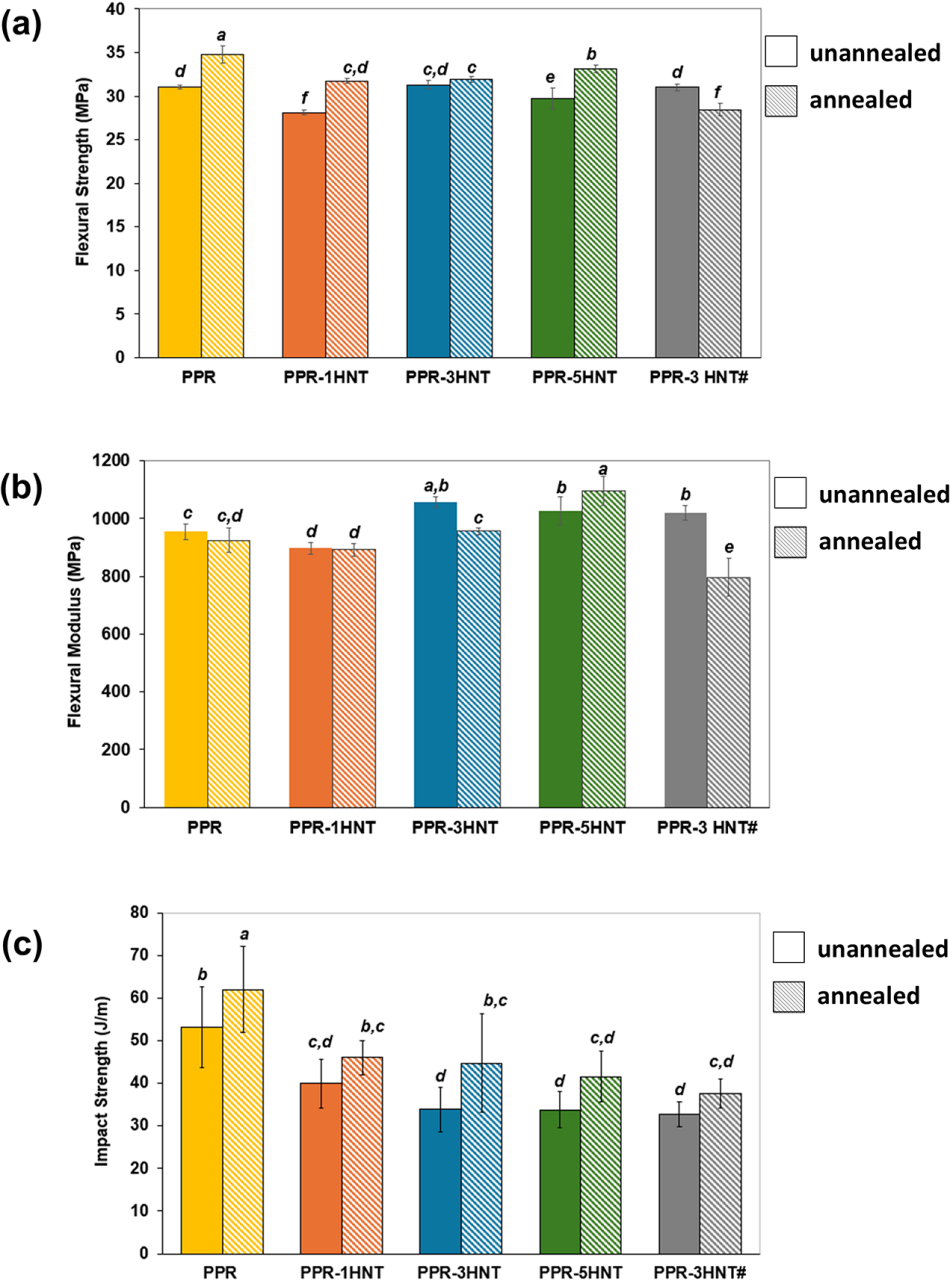
(a, b) Flexural properties and (c) Izod notched impact strength of the as-printed specimens before (solid bars) and after (hatched bars) thermal annealing treatment (Data are expressed as mean ± SD (*n* = 5), and different letters (*a*, *b*, *c* and *d*) indicate significant differences at *p* < 0.05, analyzed by one-way ANOVA with Duncan’s posthoc test).

On the other hand, the notched Izod impact strength of the FDM-printed PPR-based composite specimens (Figure 7c) showed a decreasing trend with increasing HNT content. This behavior was associated with the presence of rigid nanotubular fillers, which locally restricted matrix deformation and acted as potential stress concentration sites under impact loading. Nevertheless, the impact energy became leveled off within the examined range up to 3 wt % HNTs, indicating a limited dependence on filler content. The detrimental effect of HNT incorporation on the impact strength of the printed specimens was, however, substantially mitigated by postprinting thermal annealing at 120 °C. The improvement in impact strength observed after thermal annealing (at 120 °C for 360 min) was consistent with the DSC first-heating thermograms of the 3D-printed specimens (Figure 8), suggesting the development of a refined crystalline morphology characterized by a higher population of smaller crystals interconnected by the increased density of tie molecules, as evidenced by the DSC results. Tie molecules play a critical role in energy dissipation under impact loading by enabling effective stress transfer between crystalline domains, delaying crack initiation, and suppressing rapid crack propagation.^46^

**Figure 8:**
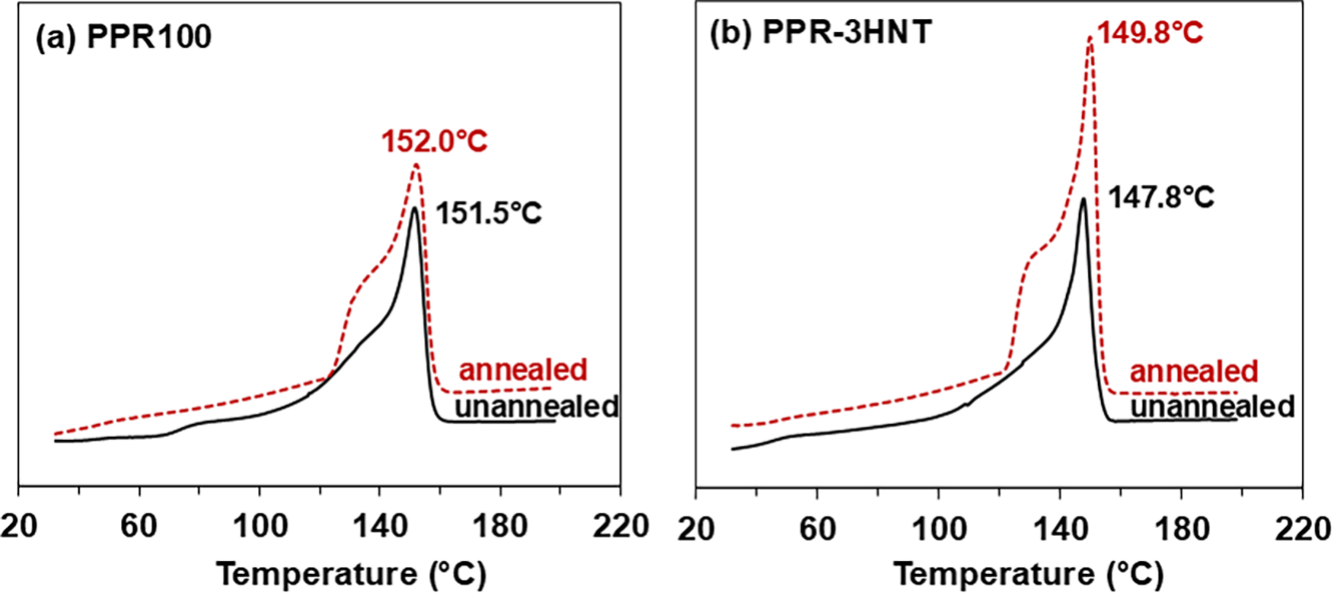
DSC first-heating thermograms of the 3D-printed specimens fabricated from (a) neat PPR100 and (b) PPR-3HNT filaments comparing the unannealed (solid black lines) and annealed (dashed red lines) conditions.

Previous studies demonstrated that the increased density of tie molecules contributed to improved toughness by promoting plastic deformation mechanisms, such as fibril stretching and crack blunting, thereby enhancing resistance to brittle fracture.^47^ Consequently, the annealing-induced increase in tie molecule density provided a plausible explanation for the partial recovery of impact strength in the HNT-filled PPR specimens, despite the presence of rigid fillers that typically promote stress concentration.

## 4 Conclusions

This study demonstrates the combined effects of halloysite nanotube (HNT) loading and dispersion, nucleation behavior, and postfabrication thermal treatment to provide an effective strategy to overcome the inherent printability limitation of semicrystalline polypropylene random copolymer (PPR), offering rational guidance for dimensionally stable and mechanically optimized PPR-HNT composite filaments for FDM applications. Incorporation of suitable amounts of HNTs improved not only the bed adhesion of printed composite filaments but also the dimensional stability of 3D-fabricated articles, while an added nucleating agent accelerated crystallization kinetics without altering the dominant crystalline phase structure. Postprinting annealing further refined the crystalline morphology, enabling partial mechanical rebalancing.

## Supplementary Material



## Notes

TEM image of HNT particles; EDX spectrum of the PPR-HNT composite; macro-morphology of the extruded PPR-HNT composite filaments; photographs captured during FDM printing on a glass build plate using (a) PPR100, (b) PPR-1HNT, (c) PPR-3HNT, (d) PPR-5HNT, and (e) PPR-3HNT^#^ filaments, illustrating differences in bed adhesion of each printing filament; and images of water droplets on the 3D article surfaces of (a) PPR100, (b) PPR-1HNT, (c) PPR-3HNT, (d) PPR-5HNT, and (e) PPR-3HNT^#^ (PDF)

## References

[1] Ngo T. D., Kashani A., Imbalzano G., Nguyen K. T., Hui D. (2018). Additive manufacturing
(3D printing): A review of materials, methods, applications and challenges. Composites, Part B.

[2] Shi Y., Faludi J. (2020). Using life cycle assessment
to determine if high utilization
is the dominant force for sustainable polymer additive manufacturing. Addit. Manuf..

[3] Anandkumar R., Babu S. R. (2019). FDM filaments with
unique segmentation since evolution:
a critical review. Prog. Addit. Manuf..

[4] Kristiawan R. B., Imaduddin F., Ariawan D., Ubaidillah, Arifin Z. (2021). A review on the fused
deposition modeling (FDM) 3D printing: Filament processing, materials,
and printing parameters. Open Eng..

[5] Dey A., Roan Eagle I. N., Yodo N. (2021). A review on filament materials for
fused filament fabrication. J. Manuf. Mater.
Process..

[6] Wang X.-Y., Gao Y., Tang Y. (2023). Sustainable
developments in polyolefin chemistry: Progress,
challenges, and outlook. Prog. Polym. Sci..

[7] Spoerk M., Holzer C., Gonzalez-Gutierrez J. (2020). Material extrusion-based
additive
manufacturing of polypropylene: A review on how to improve dimensional
inaccuracy and warpage. J. Appl. Polym. Sci..

[8] Carneiro O. S., Silva A., Gomes R. (2015). Fused deposition modeling with polypropylene. Mater. Des..

[9] Zhou Y., Zhan X., Tian C., Su K., Gong Y., Tuo X. (2025). 3D-Printing Preparation and Multi-Functional
of CNT-Filled PP/SCF
Composites. J. Appl. Polym. Sci..

[10] Bertolino M., Battegazzore D., Arrigo R., Frache A. (2021). Designing 3D printable
polypropylene: Material and process optimization through rheology. Addit. Manuf..

[11] Yost S. F., Alterio B. M., Stokes M. D., Gupta A., Vogt B. D. (2025). Enhancing
Additive Manufactured Polypropylene with Clay Nanocomposites for Both
Virgin and Recycled Resins. ACS Appl. Eng. Mater..

[12] Patiño-Almanza R., García-Méndez R. F., Rivera-Armenta J. L., Strachota A., Almendarez-Camarillo A. (2024). 3D printing
polypropylene
composites reinforced with functionalized halloysite: Balance between
stiffness and impact resistance. Polym. Compos..

[13] Zhang M., Sabatini C., Chen K., Makowka S., Hu R., Swihart M., Cheng C. (2025). Novel polymer/halloysite
composites
with high halloysite content and remarkable mechanical strength. RSC Appl. Interfaces.

[14] Libster D., Aserin A., Garti N. (2007). Advanced nucleating agents for polypropylene. Polym. Adv. Technol..

[15] Wang T. M., Xi J. T., Jin Y. (2007). A model research for prototype warp
deformation in the FDM process. Int. J. Adv.
Manuf. Technol..

[16] Jin M., Neuber C., Schmidt H.-W. (2020). Tailoring polypropylene for extrusion-based
additive manufacturing. Addit. Manuf..

[17] Jayswal A., Adanur S. (2022). Effect of heat treatment on crystallinity
and mechanical
properties of flexible structures 3D printed with fused deposition
modeling. J. Ind. Text..

[18] Eryildiz M., Kosa E., Akgun I. C., Yavuzer B. (2025). Enhancing the Mechanical
Performance of Fused Deposition Modeling-Printed Recycled Polypropylene
through Annealing Temperature, Duration, and Cooling Method. J. Mater. Eng. Perform..

[19] Moczadlo M., Chen Q., Cheng X., Smith Z. J., Caldona E. B., Advincula R. C. (2023). On the 3D printing of polypropylene and post-processing
optimization of thermomechanical properties. MRS Commun..

[20] ASTM D 7348: Standard test methods for loss on ignition (LOI) of solid combustion residues; ASTM International: West Conshohocken, PA, USA, 2021.

[21] Xiao X., Chevali V. S., Song P., He D., Wang H. (2019). Polylactide/hemp
hurd biocomposites as sustainable 3D printing feedstock. Compos. Sci. Technol..

[22] ISO 178 Plastics-Determination of Flexural Properties; ISO: Geneva, Switzerland, 2019.

[23] ISO 180 Plastics-Determination of Izod Impact Strength; ISO: Geneva, Switzerland, 2023.

[24] Liu Y., Zhao Z., Chen R., Xu X. (2023). Kinetic and thermodynamic
study of micron waste polypropylene thermal degradation. J. Polym. Sci..

[25] Wang B., Huang H.-X. (2013). Effects of halloysite nanotube orientation on crystallization
and thermal stability of polypropylene nanocomposites. Polym. Degrad. Stab..

[26] Handge U. A., Hedicke-Höchstötter K., Altstädt V. (2010). Composites
of polyamide 6 and silicate nanotubes of the mineral halloysite: influence
of molecular weight on thermal, mechanical and rheological properties. Polymer.

[27] Baheri B., Lindenberger A. L., Sharma S., Lee S. (2023). Characterization
of
linear low-density polyethylene and halloysite nanotube (LLDPE/HNT)
composites based on two-roll calendering melt fabrication. J. Appl. Polym. Sci..

[28] Lapique F., Meakin P., Feder J., Jøssang T. (2000). Relationships
between microstructure, fracture–surface morphology, and mechanical
properties in ethylene and propylene polymers and copolymers. J. Appl. Polym. Sci..

[29] Krishnaiah P., Manickam S., Ratnam C. T., Raghu M. S., Parashuram L., Prasanna Kumar S., Jeon B.-H. (2021). Mechanical, thermal and dynamic-mechanical
studies of functionalized halloysite nanotubes reinforced polypropylene
composites. Polym. Polym. Compos..

[30] Zheng H., Zeng F., Chen Z., Kang J., Chen J., Cao Y., Xiang M. (2017). Exploring
the roles of molecular structure on the β-crystallization
of polypropylene random copolymer. J. Polym.
Res..

[31] Balkaev D., Neklyudov V., Starshinova V., Stolov M., Amirova L. M., Ziyatdinova A., Amirov R. R. (2021). Novel nucleating agents for polypropylene
and modifier of its physical-mechanical properties. Mater. Today Commun..

[32] Zhao S., Cai Z., Xin Z. (2008). A highly active
novel β-nucleating agent for
isotactic polypropylene. Polymer.

[33] Li M., Li G., Zhang Z., Dai X., Mai K. (2014). Enhanced β-crystallization
in polypropylene random copolymer with a supported β-nucleating
agent. Thermochim. Acta.

[34] Kersch M., Schmidt H.-W., Altstädt V. (2016). Influence
of different beta-nucleating
agents on the morphology of isotactic polypropylene and their toughening
effectiveness. Polymer.

[35] Spoerk M., Sapkota J., Weingrill G., Fischinger T., Arbeiter F., Holzer C. (2017). Shrinkage and warpage
optimization
of expanded-perlite-filled polypropylene composites in extrusion-based
additive manufacturing. Macromol. Mater. Eng..

[36] Du M., Guo B., Wan J., Zou Q., Jia D. (2010). Effects of
halloysite
nanotubes on kinetics and activation energy of non-isothermal crystallization
of polypropylene. J. Polym. Res..

[37] Salavati M., Yousefi A. A. (2019). Polypropylene–clay
micro/nanocomposites as fused
deposition modeling filament: effect of polypropylene-g-maleic anhydride
and organo-nanoclay as chemical and physical compatibilizers. Iran. Polym. J..

[38] Liu J., Liu J. (2019). Characterization of maleic anhydride/styrene melt-grafted
random
copolypropylene and its impact on crystallization and mechanical properties
of isotactic polypropylene. J. Polym. Sci. (N.
Y. NY U. S.).

[39] Han R., Yang Q., Wang Z., Cao D., Li G., Zheng L., Peng B., Gao X., Chen G. (2022). 3D printing-enabled
self-assembling β-nucleating agent alignment: Structural evolution
and mechanical performances. Polymer.

[40] Slouf M., Pavlova E., Krejcikova S., Ostafinska A., Zhigunov A., Krzyzanek V., Sowinski P., Piorkowska E. (2018). Relations
between morphology and micromechanical properties of alpha, beta and
gamma phases of iPP. Polym. Test..

[41] Han D., Shuang S., Ye J., Ye H., Yang X., Liu Y. (2025). Polydopamine-Modified Halloysite
Nanotube Blended Sulfonated Poly
(ether Ether Ketone) Cross-Linked Composite Membrane for Fuel Cells. Energy Fuels.

[42] Moore, E. P. Polypropylene handbook: polymerization, characterization, properties, processing, applications 1996.

[43] Gilmer E.
L., Miller D., Chatham C. A., Zawaski C., Fallon J. J., Pekkanen A., Long T. E., Williams C. B., Bortner M. J. (2018). Model analysis
of feedstock behavior in fused filament fabrication: Enabling rapid
materials screening. Polymer.

[44] Vidakis N., Petousis M., Velidakis E., Tzounis L., Mountakis N., Kechagias J., Grammatikos S. (2021). Optimization of the filler concentration
on fused filament fabrication 3d printed polypropylene with titanium
dioxide nanocomposites. Materials.

[45] Qiao X., Na M., Gao P., Sun K. (2017). Halloysite nanotubes reinforced ultrahigh
molecular weight polyethylene nanocomposite films with different filler
concentration and modification. Polym. Test..

[46] Eslamian M., Bagheri R., Pircheraghi G. (2016). Co-crystallization
in ternary polyethylene
blends: tie crystal formation and mechanical properties improvement. Polym. Int..

[47] Li Y., Li Y., Han C., Yu Y., Xiao L. (2019). Morphology and properties
in the binary blends of polypropylene and propylene–ethylene
random copolymers. J. Polym. Sci..

