# Supplementary material for: Combined Effects of Halloysite Nanotubes, Nucleating Agent, and Thermal Annealing on the Printability and Mechanical Performances of 3D-Printable Polypropylene Random Copolymer-Based Composites
Source: ACS Omega. 2026 Jun 10;11(24):36033–46. doi: 10.1021/acsomega.6c03068 (PMC13295035; doi:10.1021/acsomega.6c03068)
Supplement: Supplementary file 1 [file ao6c03068_si_001.pdf]

Supporting Information

Combined Effects of Halloysite Nanotubes, Nucleating Agent, and Thermal Annealing on the Printability and Mechanical Performances of 3D-Printable Polypropylene Random Copolymer-based Composites

Boonlom Thavornyutikarn[1#*], Kawinthip Inthana[2#], Wasana Kosorn[1], Paanrapee Hongsaprapart[3], Wanida Janvikul[1*], Kalyanee Sirisinha[3,4*]

[1]*Biofunctional Materials and Devices Research Group, National Metal and Materials Technology Center, National Science and Technology Development Agency, Pathum Thani 12120, Thailand*

[2]*School of Materials Science and Innovation, Faculty of Science, Mahidol University, Nakhon Pathom 73170, Thailand*

[3]*Department of Chemistry, Faculty of Science, Mahidol University, Bangkok 10400, Thailand*

[4]*Materials and Design Unit for Medical Devices and Healthcare, Faculty of Science, Mahidol University, Bangkok 10400, Thailand*

Transmission Electron Microscopy (TEM)

The morphological characteristic of HNT particles was examined using a transmission electron microscope (3D TEM-EDS, HITACHI/HT7800, Japan), operated at an accelerating voltage of 80 kV at the magnification of 20k. In brief, HNT particles were prepared by dispersing HNT powders in ethanol (0.25 mg/mL), followed by drop-casting onto 200-mesh carbon-coated copper grids. The dimensions (average length and diameter) of at least 30 particles were measured using the ImageJ software (version 1.54d, National Institutes of Health, Bethesda, MD, USA).

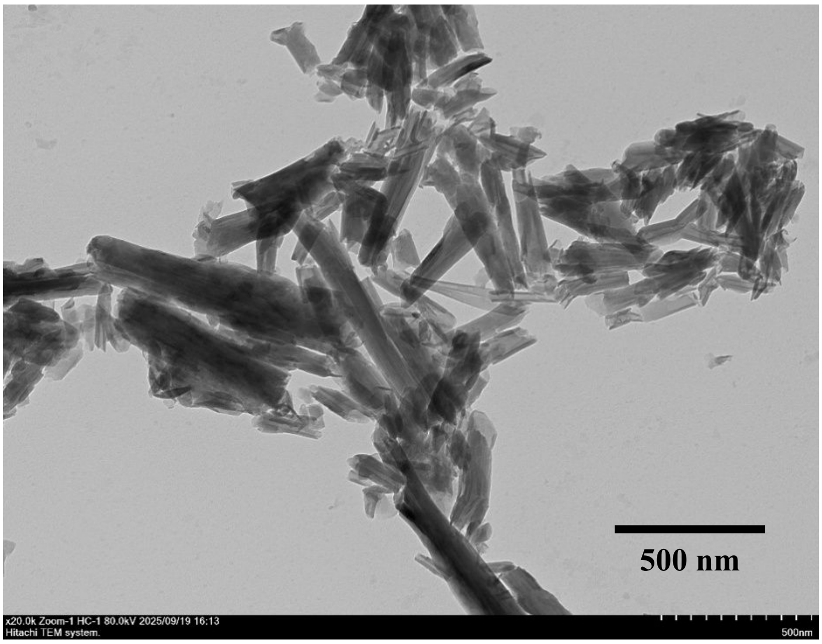

Figure S1. TEM image of HNT particles.

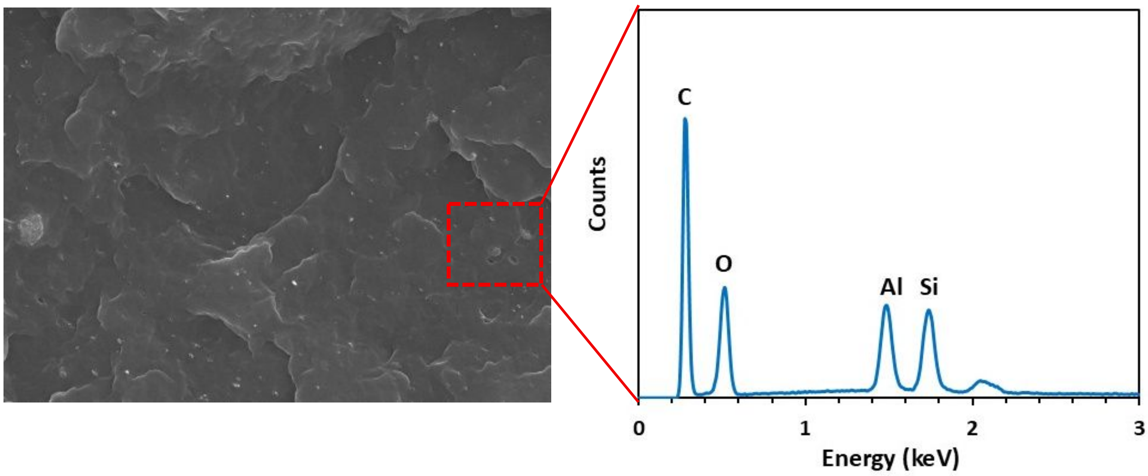

Figure S2. SEM image (left) and EDX spectrum (right) of PPR-5HNT.

Macro-morphological investigation

The macro-morphology of the extruded PPR-HNT composite filaments was examined using a digital microscope (Dino-Lite, AM4113/AD4113 series, Taiwan) at 30x magnification.

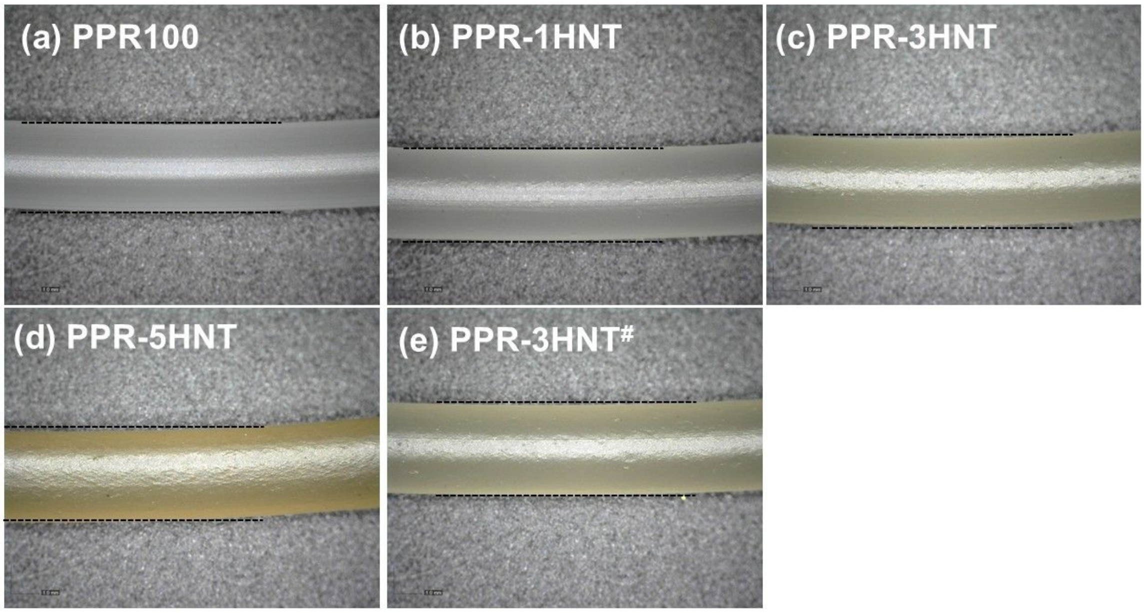

Figure S3. Stereoscopic microscopy images illustrating the surface morphology of (a) PPR100, (b) PPR-1HNT, (c) PPR-3HNT, (d) PPR-5HNT, and (e) PPR-3HNT$^{\#}$ filaments.

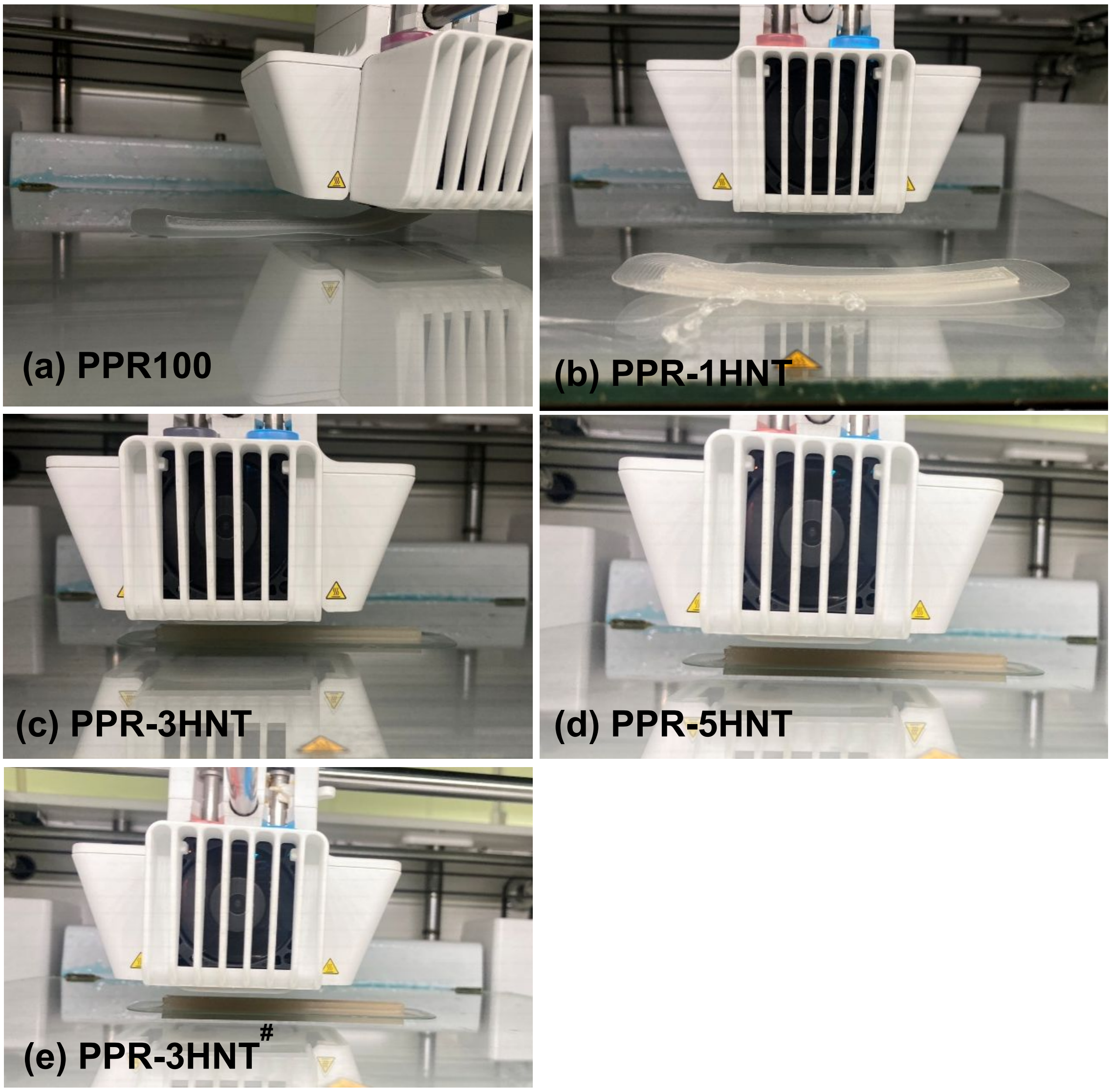

Figure S4. Photographs showing FDM printing on a glass build plate using (a) PPR100, (b) PPR-1HNT, (c) PPR-3HNT, (d) PPR-5HNT, and (e) PPR-3HNT$^{\#}$ filaments. The images were captured during printing to qualitatively illustrate differences in bed adhesion of each filament.

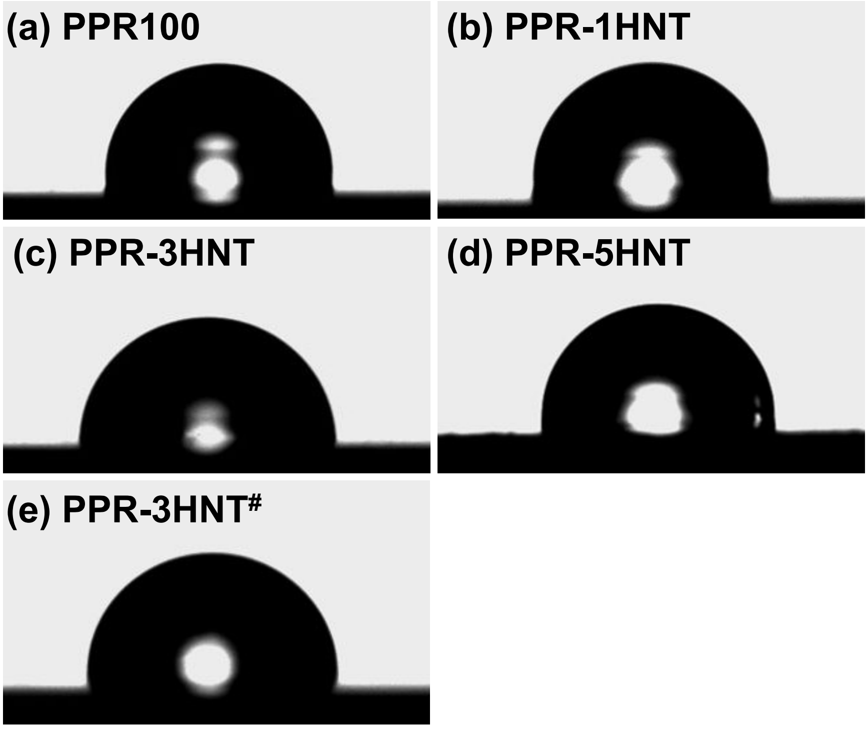

Figure S5. Images of water droplets on the 3D article surfaces of (a) PPR100, (b) PPR-1HNT, (c) PPR-3HNT, (d) PPR-5HNT, and (e) PPR-3HNT#.
